# Feto-Maternal Outcome in Pregnancy With Thrombocytopenia and Abnormal Platelet Indices

**DOI:** 10.7759/cureus.59156

**Published:** 2024-04-27

**Authors:** Dalimi Mushahary, Sheeba Marwah, Asmita Saran, Chhavi Gupta, Katyani Kumari, Sunita Malik

**Affiliations:** 1 Obstetrics and Gynecology, Vardhman Mahavir Medical College and Safdarjung Hospital, New Delhi, IND

**Keywords:** feto-maternal outcome, platelet distribution width (pdw), manual platelet count (mpc), mean platelet volume (mpv), thrombocytopenia

## Abstract

Background: Platelet count and its associated indices like mean platelet volume (MPV) and platelet distribution width (PDW) are cost-effective biomarkers that are easily accessible and have a potent role in the diagnosis and management of thrombocytopenia. Since anaemia and thrombocytopenia often go together in pregnancy, it is advisable to utilise these indices for feto-maternal benefit.

Material and methods: The study was conducted in the Department of Obstetrics and Gynaecology at a tertiary care centre in New Delhi from July 2022 to December 2023 wherein pregnant women of age 18-40 years, period of gestation >28 weeks with thrombocytopenia or abnormal platelet indices were enrolled. Women with pancytopenia, bone marrow suppression or past or current SARS-CoV-2 positive status were excluded.

Results: A total of 150 women were enrolled in the study. The mean age of study population was 25.33 ± 2.90 (range 19-34) years. Subjects were divided into three groups - Group A (mild thrombocytopenia) 24.6%, Group B (moderate thrombocytopenia) 64.6% and Group C (severe thrombocytopenia) 10.6% based on thrombocytopenia severity. Analysing the risk factors, Group C was found to have a significantly higher number of patients with anaemia (p=<0.001), fever (p=0.031), abnormal liquor volumes (p=0.004) and need for blood and platelet transfusion (p=0.077). On correlation of thrombocytopenia with abnormal platelet indices, it was observed that manual platelet count (MPC) and MPV were indirectly correlated (p=0.027). PDW was found to be directly associated with severe thrombocytopenia and indirectly associated with moderate thrombocytopenia.

Conclusion: Thrombocytopenia in pregnancy is directly correlated to factors like maternal fever and anaemia, fetal growth restriction, abnormal liquor, blood products and platelet transfusions. It was also concluded that platelet indices like PDW and MPV play an important role in predicting the feto-maternal outcome and hence timely interventions can be done to improve the same.

## Introduction

Pregnancy is associated with several changes in platelet count and platelet indices. Thrombocytopenia is the second most common hematological abnormality in pregnancy after anaemia [1]. Prevalence of thrombocytopenia is 6.6 to 11.6% (platelet count < 1,50,000/cu.mm) in pregnancy. This might be caused by accelerated platelet destruction or decreased production. Platelet indices, diagnostic and prognostic in most clinical settings are potential biomarkers of platelet activation [2]. The mean platelet volume (MPV), platelet distribution width (PDW), and manual platelet count (MPC) have good discriminatory value with the severity of the disease [1]. The PDW and MPV are the best validated and attractive indices for research in clinical settings due to their widespread availability to clinicians. According to one study [2], gestational thrombocytopenia is the commonest cause of thrombocytopenia in pregnancy with an incidence of 70% (n=100 study participants with thrombocytopenia), followed by preeclampsia (22%), hemolysis, elevated liver enzymes, low platelet count (HELLP) syndrome (4%), idiopathic thrombocytopenic purpura (ITP) (2%) and dengue (2%). In another study (where n=96 cases with thrombocytopenia) the maternal complications were placental abruption (21 cases, 21.8%), postpartum hemorrhage (14 cases, 14.6%), seven cases (7.3%) of the women were transferred to ICU and in two cases (2.1%) maternal death was seen [3]. The present study was aimed at evaluation of the feto-maternal outcome of thrombocytopenia in pregnancy and correlation of the same.

## Materials and methods

The present study was conducted in the Department of Obstetrics and Gynaecology of a tertiary care centre at New Delhi from July 2022 to December 2023. Institutional Ethical Committee Vardhman Mahavir Medical College and Safdarjung Hospital issued approval 10/43. Pregnant women between 18-40 years of age, >28 weeks gestation with thrombocytopenia (confirmed by manual platelet count <1,50,000/cu.mm) or abnormal platelet indices (mean platelet volume 6-12 fL or platelet distribution width 12-24 fL) were enrolled in the study after informed consent. Women with pancytopenia, bone marrow suppression and who were past or current SARS-CoV-2 positive status were excluded.

Formula used in the study

N ≥ ((p(1 -p))/(ME/zα) 2, where Zα was value of Z at two-sided alpha error of 5%, ME was margin of error and p was the proportion of pregnant women with thrombocytopenia.

Calculation of sample size

n>=((.0817*(1-.0817))/(.05/1.96)2 = 115.29 = 116 (approx). The final sample size was 150 patients, calculated using the software Power Analysis and Sample Size, version 8 (PASS-2008; NCSS, East Kaysville, UT, USA).

Previous literature [4] had shown that out of 1150 pregnant women, 8.17% were found to have thrombocytopenia. Taking this value as reference, the minimum required sample size with a 5% margin of error and a 5% level of significance was 116 women. To reduce the margin of error, total sample size was taken as 150 women fulfilling the eligibility criteria. After enrollment platelet indices were noted for all women in the study and they were divided into three groups: mild thrombocytopenia (Group A), moderate thrombocytopenia (Group B) and severe thrombocytopenia (Group C). All women were assessed for risk factors and materno-fetal outcomes were studied. Further as a secondary outcome thrombocytopenia was correlated with abnormal platelet indices. The study was conducted after taking institutional ethical clearance.

Statistical analysis

The data obtained were analyzed using Statistical Package for the Social Sciences (SPSS) software version 23.0 for Windows (IBM Corp., Armonk, NY, USA). Analysis was conducted using Student t test or ANOVA test for continuous variables and Pearson chi-square (χ2) test for categorical variables, whenever applicable. A two-tailed p < 0.05 was considered significant. 

## Results

The mean age of the study population was 25.33 ± 2.90 (range 19-34) years, with two-thirds (91 women out of 150) residing in rural areas. Almost half (80 out of 150) of the enrolled women were 10th pass and most of the women (149 out of 150, 99.3%) were housewives belonging to low socio-economic status (Table 1).

**Table 1:** Socio-demographic distribution of the studied pregnant women n=number of study participants

Socio-demographic variables		Frequency (n=150)	Percentage
Age Group	≤25 Years	78	52.0%
	26-30 Years	63	42.0%
	>30 Years	9	6.0%
Residence	Rural	91	60.7%
	Urban	59	39.3%
Education status	Uneducated	4	2.7%
	8 PASS	17	11.3%
	10 PASS	80	53.3%
	12 PASS	38	25.3%
	Graduate & above	11	7.3%
Occupation	Housewife	149	99.3%
	Teacher	1	0.7%
Socio-economic status	Upper class	0	0.0%
	Upper middle	1	0.7%
	Lower middle	24	16.0%
	Upper lower	75	50.0%
	Lower	50	33.3%

**Figure 1:**
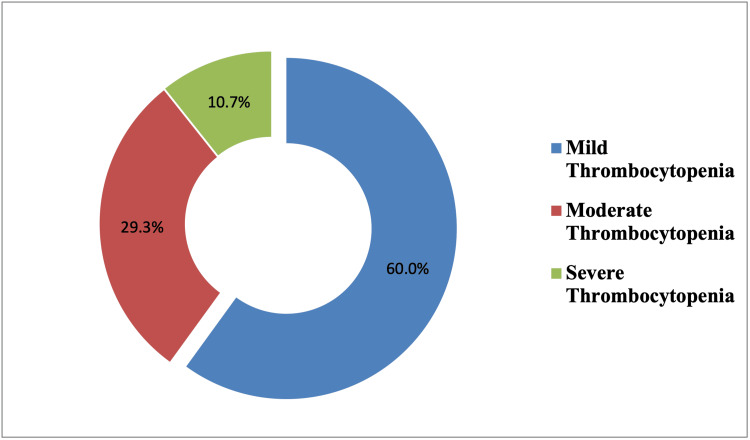
Distribution of women based on severity of thrombocytopenia %= percentage of study participants in a particular group

Most of them (89 out of 150 women, 59.3%) were primiparous with a period of gestation from 33-37 weeks. Based on the severity of thrombocytopenia subjects were divided into three groups: Group A (mild thrombocytopenia) 24.6% (90 women out of 150), Group B (moderate thrombocytopenia) 64.6% (44 out of 150) and Group C (severe thrombocytopenia) 10.6% (16 out of 150) (Figure 1).

**Figure 2:**
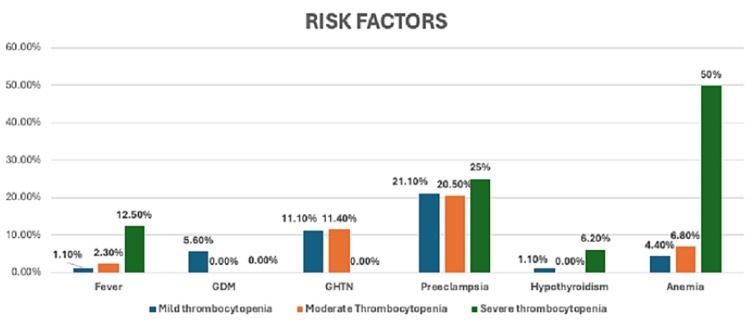
Comparison of risk factors in the three groups of thrombocytopenia %= percentage of study participants from various groups, p value <0.05 has been considered significant GDM: Gestational diabetes, GHTN: Gestational hypertension

On analysing the risk factors, it was found that fever (p=0.031) and anaemia (p=<0.001) were observed in a significantly higher number of women in Group C as compared to others. Amongst the four women with fever two were positive for dengue, one had malaria and one had pyrexia of unknown origin (PUO) (Figure 2, Table 2). Preeclampsia was also found to be increased in Group C though it was not statistically significant (p=0.077).

**Table 2:** Distribution of maternal outcome amongst the thrombocytopenia groups n= number of study participants in a particular group, p value considered significant is <0.05

Maternal Outcome	Thrombocytopenia			Pearson chi-square value	P value
	Mild (n=90)	Moderate (n=44)	Severe (n=16)		
Dengue/Malaria	1 (1.1%)	1 (2.3%)	1 (6.2%)	1.854	0.396
Anemia	7 (4.4%)	3 (6.8%)	8 (50.0%)	32.026	<0.001
Abruption	8 (8.9%)	5 (11.4%)	3 (18.8%)	1.418	0.492
Gestational Diabetes Mellitus	1 (1.1%)	1 (2.3%)	0 (0.0%)	0.545	0.761
Poly/Oligohydramnios	3 (2.2%)	0 (0.0%)	3 (18.8%)	11.003	0.004
Premature rupture of membranes (PROM)	22 (24.4%)	10 (22.7%)	1 (6.2%)	2.640	0.267
Postpartum hemorrhage (PPH)	6 (6.7%)	4 (9.1%)	1 (6.2%)	0.287	0.867
Blood transfusion and platelet transfusion	19 (21.1%)	18 (40.9%)	10 (62.5%)	13.470	0.001

When discerning the maternal outcome in each of the participants, abnormal liquor volumes (p=0.004) and need for blood and platelet transfusion (P=0.001) were present significantly higher in Group C. Also women in Group C had increased incidence of abruption (three out of 16, 18.8%), postpartum haemorrhage (PPH) (one out of 16, 6.2%), though not statistically significant (Table 2).

**Figure 3:**
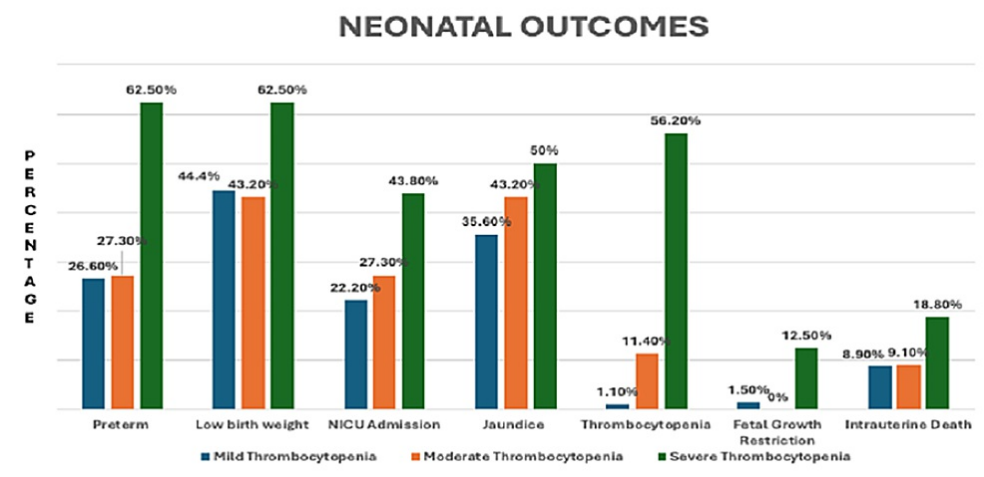
Distribution of neonatal outcome amongst thrombocytopenia groups % = % of study participants in a particular group NICU: neonatal intensive care unit

It was found that preterm births (p=0.011), neonatal thrombocytopenia (p<0.001) and fetal growth restriction (p=0.027) were reported insignificantly higher in women of Group C. Low birth weight, neonatal intensive care unit (NICU) admission and jaundice were also reported insignificantly higher in women of Group C in the present study. Stillbirth was also noted in 18.8% of cases (three out of 16 women in Group C) (Figure 3).

On correlating thrombocytopenia with abnormal platelet indices, it was observed that MPC and MPV were significantly inversely associated (p=0.012) with thrombocytopenia. PDW was positively correlated with severe thrombocytopenia (p>0.05) but was inversely correlated with moderate thrombocytopenia (p<0.001) (Table 3).

**Table 3:** Correlation between platelet parameters in studied pregnant women p value considered significant is <0.05 MPV: mean platelet volume, PDW: platelet distribution width, MPC: manual platelet count

Platelet parameters	Mild to Moderate		Severe	
	Pearson Correlation	Sig. (2-tailed)	Pearson Correlation	Sig. (2-tailed)
MPC vs. MPV	-0.217^*^	0.012	-0.312	0.239
MPC vs. PDW	-0.284^**^	<0.001	0.103	0.704

## Discussion

This study was conducted to evaluate the risk factors of thrombocytopenia and abnormal platelet indices in pregnancy and their effect on feto-maternal outcome. In the present study, the mean age was 25.33±2.90 years and out of 150 women, 91 (60.7% of enrolled women) were living in rural areas. These observations were comparable to the study conducted by Sojitra et al. [5]. Whereas in another study conducted by Gebreweld et al., the mean age was reported higher i.e. 27.3 ± 4.48 years but the age range of participants was similar to our study [6]. They also noted that the majority of the women (118 out of 284 women, 41.5%) were in the age range of 26-30 years and were urban residents (261 out of 284 women, 91.9%). Belayneh et al. reported a mean age of 28.9±5.9 years in their study and 119 out of 193 (61.7%) women were less than 30 years of age [7]. This was in parallel to the young age group of women who seek antenatal care in the hospital. In the existent study, the majority (59.3%, n=150) of the women were primigravida which is comparable to the study by Grum et al. [8] which included 60% (n=291) of primigravida women and in contrast with earlier studies by Sojitra et al. (multigravida 60%, n=25) [5]. This is because our study was conducted in a tertiary care hospital, having maximum load of primigravida and high-risk women for delivery and management. 

The mean gestational age of women at delivery was 38 weeks in this study which was comparable to an earlier study by Sojitra et al. [5]. Pirjani et al. [9] also reported that the majority of their participant women delivered at term. Further, the period of gestation was significantly lower in the patients of severe thrombocytopenia (p=0.014) causing preterm delivery which was also observed by Vishwekar et al. [10]. This may be due to severe thrombocytopenia being associated with preeclampsia which leads to inflammation of the placenta and membranes, predisposing to preterm labour.

In the present study, 64.7% (n=150) of women had moderate thrombocytopenia, 24.7% (n=150) had mild thrombocytopenia and 10.7% (n=150) were cases of severe thrombocytopenia. In comparison, Belayneh et al. [7] reported that among the thrombocytopenic pregnant mothers (n=26 women) 73.3% had mild, 15.38% (n=26 women) moderate and 11.54% (n=26 women) had severe thrombocytopenia.

In this study when the risk factors were also considered then fever (p=0.031) and anemia (p<0.001) were prevalent in a significantly higher number of women with severe thrombocytopenia which was comparable to other studies [7]. This was because of the inflammation caused by various medical conditions like dengue and malaria leading to excessive platelet recruitment to the site of inflammation. Anemia was a significant finding in this study which may be due to high prevalence of nutritional deficiencies in India along with the study cohort neglecting intake of iron and folic acid supplement during pregnancy.

In our study, around 21.33% (n=150) of women had preeclampsia, followed by hypertension and anemia (10.0% each, n=150) which was comparable to the study by Padmawar et al. [2]. This is in contrast to the work of Qureshi et al. in which preeclampsia was observed in 14.6% (n=86) cases [3]. The variation may be due to differences in timing of presentation on which the diagnosis is largely dependent, besides severity of thrombocytopenia and the association with other abnormalities. 

In the present study fetal growth restriction (12.5%, n=16) and oligohydramnios (18.8%, n=16) were present in a significantly higher number of patients suffering from severe thrombocytopenia (p<0.05). Both hypoproduction and ineffective thrombopoiesis are the underlying pathological mechanisms in megaloblastic thrombocytopenia as evidenced by the marrow findings and platelet indices. Platelet indices are of significant discriminative value in differentiating the various causes of thrombocytopenia [11]. Qureshi et al. showed placental abruption in 21.8% (n=86), postpartum hemorrhage in 14.6% (n=86) and maternal mortality in 2.1% (n=86) of these women [3]. Chauhan et al. [12] reported that pregnancy-induced hypertension (PIH) was associated with 26.2% (n=65) of thrombocytopenic women. Among the 17 women who had pregnancy-induced hypertension, the majority of them had gestational hypertension in 64.9% followed by pre-eclampsia in 23.5%. Severe pre-eclampsia was noted in one (5.8%) woman. Only one had eclampsia (5.8%). A study by Sultana et al. [13] showed poor maternal outcomes in 11% (n=100) cases due to HELLP syndrome, postpartum haemorrhage, and maternal death. 

In this study, blood transfusion (BT) and platelet transfusion (PT) were needed in a significantly higher number of patients suffering from severe thrombocytopenia (p<0.05). This is in contrast to another study where requirement of blood and platelet transfusion was lower [5]. Our study showed that preterm birth (p=0.011) and neonatal thrombocytopenia (p<0.001) were observed in a significantly higher number of neonates born to mothers with severe thrombocytopenia. It was seen that most pregnant (10 out of 16 women, 62.5%) women who went into preterm labour had severe thrombocytopenia. This can be explained by the fact that in preterm labour, inflammatory cytokines are increased leading to platelet activation.

An increase in PDW has been associated with an increase in platelet activation which might contribute to the hypercoagulability associated with pregnancy. PDW reflects anisocytosis (difference in size of platelets), which may occur due to increased production of platelets in bone marrow resulting in large and varied size platelets. Uncontrolled platelet activation and aggregation are expected in non-thrombocytopenic pregnancies as well [2]. It can lead to increased platelet consumption though compensated by bone marrow. Increased PDW reflecting increased bone marrow turnover even in groups having normal platelet count suggests platelet activation and aggregation in non-thrombocytopenic conditions in pregnancy as well. Probably, this is the reason for increased PDW even in normal controls as pregnancy itself is a hypercoagulable state [11]. 

The inferences drawn from our study cannot be extrapolated to the entire population size since it was a single-centre study with a small sample size, conducted in the backdrop of the coronavirus disease 2019 (COVID-19) pandemic, hence this becomes a limiting factor for the present study.

## Conclusions

On correlation of maternal outcome with the severity of thrombocytopenia, anemia, foetal growth restriction, abnormal liquor volume and need for blood and platelet transfusion were present significantly in women with severe thrombocytopenia. Also, preterm birth and neonatal thrombocytopenia were noted significantly in higher number of infants born to mothers with severe thrombocytopenia. Determination of these platelet indices can thus signal timely intervention to prevent future complications. Hence, serial estimation of platelet indices in pregnant women is advocated and emphasized upon across all health facilities to decrease the feto-maternal morbidity. Further, studies with larger sample sizes are required to validate the conclusions drawn by the present research.
